# Characterizing diet quality indicators and their demographic determinants among adults: a population-based study in Northern Ghana

**DOI:** 10.3389/fnut.2025.1633285

**Published:** 2025-09-02

**Authors:** Paula Malebna Kolbila, Musah Abdul-Samed, Seidu Rahama, Afia Aboabea Apau-Tete, Bruce A. Abugri, Bright Yammaha Amoore, Patience K. Gaa, Victor Mogre

**Affiliations:** ^1^Department of Community and Preventive Medicine, School of Medicine, University for Development Studies, Tamale, Ghana; ^2^Department of Nutritional Sciences, School of Allied Health Sciences, University for Development Studies, Tamale, Ghana; ^3^Department of Dietetics, School of Allied Health Sciences, University for Development Studies, Tamale, Ghana; ^4^Department of Health Professions Education and Innovative Learning, School of Medicine, University for Development Studies, Tamale, Ghana

**Keywords:** diet, food habits, healthy eating, non-communicable diseases, Ghana

## Abstract

**Objectives:**

Unhealthy eating patterns increase the risk of non-communicable diseases (NCDs), such as type 2 diabetes, heart disease, and cancer. A healthy diet should provide energy and nutrients for growth, maintenance, activity, and infection prevention. Global indicators include dietary diversity score, following recommended food groups, and NCD-protective consumption. We assessed dietary patterns and associated demographic factors among adult Ghanaians from northern Ghana using indicators aligned with global recommendations for healthy eating.

**Methods:**

A cross-sectional design was employed, utilizing a diet quality questionnaire (DQ-Q) to evaluate five indicators: food group diversity score (FGDS), all-5 recommended food groups, NCD-protect scores, NCD-risk scores, and global dietary recommendation (GDR) scores. Data analysis incorporated Spearman's rho, Mann-Whitney, Kruskal-Wallis tests, and multiple linear regression to explore associations.

**Results:**

A total of 842 participants were recruited. In which 96.4% consumed starchy staples, over 90% ate vegetables, and 70% included fruits in their diet. Mean (SD) scores for FGDS, NCD-protect, World Health Organization (WHO) all-five food groups, NCD-risk, and GDR were 10, 9, 5, 8, and 18, respectively. A moderately positive correlation was observed between NCD-protect scores and FGDS (*r* = 0.763, *P* = 0.001), as well as with WHO all-five groups (*r* = 0.688, *P* < 0.001). Higher education was the strongest predictor of better diet quality—those with secondary education ate more protective foods, had greater dietary diversity, and better WHO-recommended food adherence—while Mole-Dagbani/Gonja ethnicity was consistently associated with poorer diet quality; married/cohabiting status modestly increased both protective and risk food consumption, and each additional year of age slightly reduced NCD-risk food intake.

**Conclusion:**

Consumption of staple foods was widespread. Vegetables were consumed frequently, but not fruits. Diets were only moderately diverse, and dietary patterns reflecting NCD-risk factors were prevalent. Dietary patterns reflecting NCD-risk factors were widespread. Ethnicity, marital status, and employment status significantly predicted diet quality indicators, informing future dietary guidelines.

## Introduction

Globally, an unwholesome diet pattern presents a substantial hazard for non-communicable diseases (NCDs), such as type 2 diabetes, heart-related issues, and various forms of cancer (1, 2). Unhealthy diets account for between 20 and 25% of all deaths in adults (3–7). A quality diet has the potential to prevent malnutrition and provide energy and all essential nutrients to support body maintenance, growth, physical activity, and protect against infections (8). The World Health Organization (WHO) recommendations for a healthy diet include regular consumption of fruits, vegetables, legumes, nuts, and grains. Furthermore, a healthy diet should consist of cutting down on salt, sugar, and fats, specifically choosing unsaturated fats over saturated fats (9). In addition, the World Cancer Research Fund recommends limiting the consumption of red meats and avoiding processed meats (10–12).

Notwithstanding the fact that specific foods and nutrients are critical in promoting good health, overall dietary patterns are significant determinants of health (13), given that different foods interact through synergistic relations to produce complementary effects. It is, thus, imperative to evaluate dietary patterns at the population level to inform intervention approaches to guide policy decisions.

Globally, dietary patterns are increasingly becoming unhealthy.

According to the 2021 Global Nutrition Report, consumption of healthy foods has slightly increased, mostly from fruits, vegetables, and whole grains, but consumption of legumes and sugar-sweetened beverages has decreased. According to the 2015 Global Dietary Database, unhealthy dietary patterns scored 51, while healthy ones had an average score of 48, showing a diet pattern that is largely unhealthy. All seven world regions exceeded the maximum target for added sugars and red meats, and none of them met the EAT-Lancet targets for fruits, non-starchy vegetables, beans, legumes, nuts, seeds, or whole grains (6, 14, 15). Regional variations exist, with sub-Saharan Africa reporting a high prevalence of unhealthy dietary patterns. In a systematic analysis of global data, the 2015 Global Dietary Database, Sub-Saharan Africa had the highest unhealthy dietary pattern scores but the lowest healthy dietary pattern scores (14). In 2018, while fruit and vegetable consumption was 59% below recommended intake in Africa, Europe, and Northern America had lower rates at 41 and 56%, respectively (6). Also, the consumption of red and processed meats was doubled in Africa, while fruit and vegetable consumption in 2018 was 59% below recommended intake in Africa, but also 41 and 56% below recommendations in Europe and Northern America, respectively (6).

These shifts in dietary patterns have been identified as the “nutrition transition,” spurred by factors like heightened manufacturing of processed foods, rapid urbanization, and evolving lifestyles (16). This shift, coupled with a more inactive way of life, has played a role in the emergence of obesity and other persistent health issues in developing countries (17).

A few studies have previously investigated the dietary patterns of Ghanaian adults (18–21). Through the Research on Obesity and Diabetes among African Migrants (RODAM) study, Galbette et al. investigated the dietary patterns among Ghanaian migrants in Europe and their compatriots from the Ashanti region of Ghana, in which they found that diets were either mainly starchy foods and/or animal-based products (18). Frank et al. (19) evaluated dietary patterns in urban Ghana and risk of type 2 diabetes in a hospital-based case-control study in Kumasi in which they identified two diet patterns: purchase' dietary pattern (characterized by high intakes of sweets, rice, meat, fruits, and vegetables) and a “traditional” dietary pattern (characterized by high intakes of fruits, plantain, green leafy vegetables, fish, fermented maize products, and palm oil). A nationwide survey on diet quality published by the Global Diet Quality Project for Ghanaian women and men aged 15 years and older found that only 23% of women and 25% men were consuming at least 400 g of fruits and vegetables. On the other hand, 46% of women and 47% of men took sugar-sweetened beverages (20). None of the studies characterize diet patterns according to the WHO recommendations for a healthy diet. Except for the single nationwide study, the two other studies were conducted in Southern Ghana, specifically Kumasi. Also, two studies were community-based, and the remaining one was hospital-based. Thus, data regarding the characterization of the diet pattern of Ghanaians in line with the WHO recommendations of a healthy diet (especially from northern Ghana) and at the population level is limited.

Characterization or indicators of a healthy diet are necessary in order to monitor a key public health risk factor (10). In meeting the WHO recommendations, the indicators of a quality diet should reflect the protection of health against diet-related NCDs, as well as promoting nutrient adequacy. The current study intends to describe diet quality indicators at the population level using the metrics described by Herforth et al. (10), reflecting the WHO global recommendations of a healthy diet. Thus, we aim to evaluate diet quality indicators and associated demographic factors among adult Ghanaians from northern Ghana. The evidence generated will contribute to forming agricultural policies to promote the production of healthy foods; the development of policies and national dietary guidelines; and the development of culturally sensitive interventions to promote awareness and the consumption of healthy foods.

## Methods

### Study area

This study was conducted in the Tamale metropolis from April through June 2023. The Tamale Metropolitan Assembly (TMA) is among the 261 Metropolitan, Municipal, and District Assemblies (MMDAs) in Ghana. In 2004, TMA was given metropolis status. The Tamale Metropolitan Area is positioned in the central portion of the northern Region and is elevated at about 180 meters above sea level. Tamale metropolis shares boundaries with East Ganja Municipality to the South, Yendi Municipal Assembly to the East, Savelugu Municipality to the North, Tolon District to the West and Central Gonja District to the South-West. According to the 2021 population and housing census, the Tamale metropolis is made up of 112 suburbs and has a population of 374,744 people, of which 50.6% are female. TMA was chosen for the current study due to its rising urbanization, nutrition transition, and the double burden of malnutrition. The metropolis exhibits hallmarks of a rapidly developing city—urbanization, globalization, and spatial expansion—evidenced by supermarket growth and the entry of fast-food restaurants such as the international Kentucky Fried Chicken (KFC) and local franchise Chicken Republic, alongside rising slum development and urban poverty (22). Tamale can be described as a true representation of the triple burden of malnutrition, given that NCDs are rising while infectious diseases, undernutrition, and micronutrient deficiencies are endemic.

### Study design, participants, inclusion, and exclusion criteria

A community-based cross-sectional design was utilized. All adults living in the Tamale metropolis over the age of 18 years made up the target group. Participants were eligible for inclusion if they had lived in the community for at least 6 months. Participants were excluded if they were mentally challenged, indisposed or did not consent to participate in the study. Mentally ill individuals may struggle with informed consent due to impaired cognitive function, affecting their understanding of the study's purpose, procedures, risks, and benefits.

### Determination of sample size

The sample size was determined using the following Cochcran's formula (23):


$$\begin{array}{l} N=\;\frac{z^{2}\;\left(P\right)\left(1-P\right)\left(DEFF\right)}{d^{2}} \end{array}$$

N = Sample Size

Z = z-score of the confidence level (95%) = 1.96

ME = margin of error (5%) = 0.05

p = estimated prevalence. Due to the unavailability of data on the prevalence of diet quality in the Tamale metropolis, we assumed 50% (0.5) prevalence. We relied on previously reported prevalence estimates (1%−48%) for consumption of NCD-risk foods—such as sugar-sweetened beverages (sodas, energy drinks), deep-fried snacks (French fries, plantain chips), processed meats (sausages, cold cuts), and high-salt packaged foods—to contextualize our findings (20). To cater for variability, we adopted 50% which is assumed to be a conservative value (producing the largest sample size possible given the other parameters held constant) (24).

DEFF = design effect due to our adoption of a cluster sampling approach. DEFF is a measure of the homogeneity within a cluster and the variability between clusters, describing how large the sampling variance (square of the standard error) is for the cluster sample compared to a simple random sample of the same size (Micronutrient survey toolkit, https://mnsurvey.nutritionintl.org/categories/13). We assumed a design effect (DEFF) of 2.0 to account for the extra variance introduced by our two-stage cluster sampling (suburbs → households). In similar population-based nutrition and dietary surveys in Ghana and elsewhere in sub-Saharan Africa (25), DEFF values for dietary and anthropometric indicators typically range from 1.5 to 2.5. By using 2.0, we adopt a conservative default—recommended by WHO and other survey-methodology guidelines—so that the sample size remains adequately powered to detect meaningful differences even in the presence of within-cluster homogeneity (26).

After accounting for the estimated prevalence, a design effect of 2.0, and inflating by 10% to compensate for potential non-response, the calculated minimum sample size required for this study was 845 participants.

### Sampling method

The cluster sampling technique was used to select the households. The 112 suburbs in the Tamale metropolis represented clusters. An unbiased random sample of 10 communities was drawn from the full list of 112 using Excel's random-number generator to ensure each community had an equal chance of selection. We selected 10 communities to balance statistical precision (≈85 respondents per cluster ensures adequate power with our design effect), logistical feasibility (field teams can reliably survey and supervise data collection at 10 sites), and geographic representativeness. Given the large nature of the selected communities, segments were created based on natural landmarks and public places, such as roads, markets, schools, churches, mosques, and temples. One segment was chosen through simple random sampling by the research assistants (RAs). Given that the households in the selected suburbs of the metropolis are not listed in a defined geometric setting and the impossibility of making a list of households, a modified Expanded Programme on Immunization approach was adopted to select the households for the study (24). Using this approach, RAs identified a location near the center of the segment/community; defined a random direction through a bottle or pen toss and a random household along the chosen direction pointing outwards from the center of the community to its boundary (27). In subsequent steps, which were carried out iteratively, the closest household (door to door) to that determined in the previous step was chosen and checked for compliance with the inclusion criteria (27). The iterations were repeated until the required number of households was surveyed. This is a valid approach (28) and has enabled WHO and UNICEF to evaluate the coverage of their childhood immunization programs and has also been adapted to measure nutritional status (29).

### Data collection procedure

Within the randomly selected communities, we carried out community entry and engagement activities with community leaders for the purposes of information dissemination regarding the study and recruitment of households and participants. Data were collected by trained RAs. These trained RAs visited each randomly selected community segment and, using pre-assigned random routes, selected households at random for the survey. Within the selected households, potential participants were informed of the significance of the study following a brief introduction and statement of purpose and were taken through all informed consent procedures, and those who agreed to participate were given a form to sign prior to participation in the study. Data were collected using a questionnaire. All informed consent processes and data collection were conducted in the homes of the participants. Although the questionnaire was designed in English, it was read in Dagbani and Twi for those who did not speak nor read in English. Dagbani is the dominant language in Tamale, spoken by the Dagomba majority and used broadly in commerce and daily interaction. Twi serves as a regional lingua franca among migrant and trading communities in the metropolis, reflecting its status as one of Ghana's most widely spoken indigenous languages.

Each participant took about 5–10 min to complete the questionnaire. The questionnaire was piloted on seven participants to evaluate the questions' wording and order, the feasibility of the data collection design, and any other potential problems with the questionnaire. Any difficulties encountered while administering the questionnaire were properly resolved.

### Data collection methods

The study used a structured questionnaire to obtain its data. The questionnaire assessed participants' socio-demographic characteristics, including age, gender, marital status, ethnicity, and educational level. Diet quality was assessed using the diet quality questionnaire (DQ-Q), designed and previously validated for the Ghanaian population (10, 20). We adopted the tool completely without alterations to it. The DQ-Q contained 29 food groups consisting of a set of yes/no questions about the consumption of the food groups during the previous day or night. Respondents were asked whether they consumed any of up to seven sentinel foods per question, which are the most commonly consumed food items in each food group in the Ghanaian setting (20, 30). Purposely, the food groups are designed to reflect a holistic diet quality. The DQ-Q is designed for monitoring diet quality at the population level and can be used for characterization of dietary patterns (31). It is designed to assess nutrient adequacy and dietary patterns in relation to NCD risk, consumption of diets in relation to the nutrition transition and sustainability (31). It is a standardized data collection tool and can be used to compute indicators that capture health-protective patterns and unhealthy food intake, aligned with dietary benchmarks expressly intended for global application. Every individual food and beverage item is allocated to one of the 29 food groups within the DQ-Q. This categorization assists in recognizing the most frequently consumed items within each group. Subsequently, individuals are grouped based on whether they consumed at least one item within each specific food group (assigned a value of 1) or did not consume any items (assigned a value of 0) (10, 31). This study uses five diet quality indicators based on dietary guidelines from the World Health Organization (WHO) and the Food and Agriculture Organization (FAO), which emphasize the importance of nutrient-dense foods and balanced eating patterns in promoting overall health and preventing NCDs. The indicators include food group diversity scores (FGDS), NCD-Protect Scores, all-5 WHO Recommended Food Group Scores, NCD-Risk Scores, and global dietary recommendation (GDR) scores. The selection of these indicators is based on the understanding that a balanced diet rich in nutrient-dense foods is key to preventing diet-related NCDs. This framework aligns with the concept of a healthy diet, which prioritizes diversity, quality, and moderation. These indicators are consistent with the understanding of a healthy diet, which emphasizes nutrient adequacy, disease prevention, and adhering to global recommendations, such as those from WHO, which advocate for diets rich in plant-based foods and low in processed items.

#### Food group diversity score (FGDS)

The FGDS is a semi-continuous score (0–10) of the whole population. It corresponds to the 10 food groups of the minimum dietary diversity for Women of Reproductive Age (MDD-W) that has been previously validated for micronutrient adequacy in women of reproductive age in low- and middle-income countries (10, 31, 32). Scores range between 0 and 10, with the higher the score, the higher the likelihood of nutrient adequacy. The 10 food groups were grains, white roots and tubers, and plantains, pulses (beans, peas, and lentils), nuts and seeds, dairy, meat, poultry and fish, eggs, dark green leafy vegetables, other vitamin A-rich fruits and vegetables, and other vegetables.

#### Consumed all-five (all-5) recommended food groups

Refers to the frequency and proportion of the participants consuming all-five (all-5) food groups usually recommended in food-based dietary guidelines for daily consumption (10, 31). These are fruits, vegetables, pulses, nuts, or seeds; animal-source foods; and starchy staples. A score of 5 indicates minimal adherence to dietary guidelines. A score of < 5 indicates that not all five recommended food groups were consumed. all-5 was expressed as the frequency and percentage of the population consuming all five recommended food groups the previous day or night.

#### NCD protect score

This is an indicator of dietary factors protective against NCDs as recommended by the WHO relating to the consumption of at least 400 g of fruits and vegetables in a day, whole grains, pulses and nuts or seeds and at least 25 g of fiber per day (10, 31). NCD protect score ranges between 0 and 9 and reflects adherence to GDRs on healthy components of the diet. It reflects the consumption of food from nine healthy food groups during the past day and night: whole grains, pulses, nuts, and seeds, vitamin A-rich orange vegetables, dark green leafy vegetables, other vegetables, vitamin A-rich fruits, citrus, and other fruits (10, 31). A higher score indicates the consumption of more health-promoting foods in the diet (31). It is reported as an average score for the participants.

#### NCD-risk score

This is an indicator of dietary factors for NCDs as recommended by the WHO and derived from the WHO International Agency for Research on Cancer (10, 31). It is also a proxy measure for the consumption of ultra-processed foods. A higher NCD-risk score is closely related to higher ultra-processed food consumption. The NCD-risk score is a score with a range from 0 to 9 and reflects adherence to GDRs on components of the diet to limit or avoid. A higher score indicates higher consumption of foods and drinks to avoid or limit, and correlates negatively with meeting GDRs. The NCD-risk score is based on food consumption from eight food groups to limit or avoid during the past day and night (one food group, processed meat, is double weighted). These food groups are: soft drinks (sodas), baked/grain-based sweets, other sweets, processed meat, unprocessed red meat, deep fried foods, fast food and instant noodles, and packaged ultra-processed salty snacks.

#### Global dietary recommendation (GDR) score

Ranging between 0 and 18, the GDR score indicates adherence to GDRs comprising dietary factors protective against NCDs (10, 31). The higher the score, the higher the likelihood of meeting the global recommendations for food group consumption during the past day and night. It was calculated based on the following: (NCD-Protect—NCD-Risk) and reported as an average score for the population (10). A score of 10 or more corresponds to meeting at least half of the recommendations (10).

The DQ-Q and how the indicators were derived can be found in Ref. (33).

### Data analysis

The collected data were encoded, processed, and analyzed utilizing the Windows version of the IBM Statistical Package for Social Science (SPSS) software version 26, with a significance level set at 5%. Frequencies and percentages were used to describe five demographic characteristics: sex, ethnicity, educational level, marital status, and employment status. Means and standard deviations were used to describe age. Frequency tables, percentages, and bar charts were used to describe and present the proportion of participants consuming the food groups that constituted each of the five diet quality indicators. The scores of the five diet quality indicators were assumed to be semi-continuous, and as such, normality of the data was assessed using the Shapiro and Kolmogorov-Smirnov test, which was found not to be normally distributed. Spearman's rho test was used to determine the relationship between age (a continuous variable) and the five diet quality indicators. Mann-Whitney *U*-test was adopted to determine the association between the scores of the five diet quality indicators and sex (male vs. female), and employment status (employed vs. unemployed). To determine the association between the scores of the five diet quality indicators and socio-demographic variables that had more than two levels (i.e., ethnicity, level of education, and marital status), Kruskal-Wallis test was used. Multiple linear regression analysis was performed, in which B coefficients were reported at a 95% confidence interval. To evaluate the independent relationship between each socio-demographic characteristic and our five diet-quality measures, we fitted multivariable regression models that simultaneously adjusted for age (in years, continuous), sex (male vs. female), highest educational attainment (none, primary, secondary, or tertiary), employment status (employed vs. unemployed), ethnicity (categorical), and marital status (married vs. unmarried). By including all of these covariates in the same model, we were able to estimate the unique contribution of each factor to diet quality while holding the others constant.

### Ethical considerations

The University for Development Studies Institutional Review Committee granted ethical approval for this study (Reference number: UDS/RB/030/23). Since participation in this work was optional, participants were not in any way coerced into taking part. Second, participants were included based on their informed consent, which was achieved by giving them sufficient information and assurances regarding participating in the study so they could decide whether to do so. They were told they could stop filling out the questionnaire at any time while the interview was going on. Furthermore, offensive, racist, and insulting language was not used in the questionnaire's design. Finally, participants' identities and the confidentiality of their data were maintained. Each participant was assigned a unique study ID; all names and addresses were stripped from the analysis dataset. Consent forms and ID-key files are kept separately in locked storage, and electronic data resides on an encrypted, password-protected drive accessible only to core staff. Only aggregated results are ever reported.

## Results

In all, 846 questionnaires were administered, of which 842 were found to be complete and included in the analyses (completion rate = 99.5%). Socio-demographic characteristics of the participants are presented in Table 1. Participants had a mean age of 31.55 (9.99) years, were frequently married (49.9%, *n* = 419), Muslims (60.1%, *n* = 503), and belonged to the Mole-Dagbani ethnicity (79.8%, *n* = 672).

**Table 1:** Socio-demographic characteristics of the participants.

**Variable**	**Frequency**	**Percentage (%)**
**Sex**		
Female	462	55.4
Male	372	44.6
**Ethnicity**		
Mole-Dagbani	672	79.8
Akan/Ewe/Ga	119	14.1
Others	51	6.1
**Educational level**		
No education	138	16.4
Primary	62	7.4
Middle/JHS	86	10.2
Secondary	156	18.5
Tertiary	400	47.5
**Marital status**		
Married/cohabiting/living with a partner	419	49.9
Divorced/separated/widowed	34	4.0
Never married/single	387	46.1
**Employment status**		
Employed	517	62.7
Unemployed	307	37.3

JHS, junior high school.

### Food group diversity score

As shown in Table 2, consumption of starchy staples, grains, roots, and tubers was consumed by 96.4% of the participants, 88.2% ate meat, poultry, and fish, and 48.6% ate dark green leafy vegetables. Participants had a mean food group diversity score of 6.02 (2.04) out a total score of 10.

**Table 2:** Proportion of participants consuming at least one food item from 10 food groups during the previous day or night.

**Variable**	**Frequency**	**Percentage (%)**
Starchy staples, grains, roots, and tubers	812	96.4
Pulses (beans, peas, and lentils)	355	43.4
Dark green leafy vegetables	409	48.6
Other vegetables	709	84.2
Other vitamin A-rich fruits and vegetables	378	44.9
Other fruits	410	48.7
Eggs consumption	328	39.4
Meat, poultry, and fish	743	88.2
Nuts and seeds	504	61.1
Dairy	417	49.5

**Figure 1:**
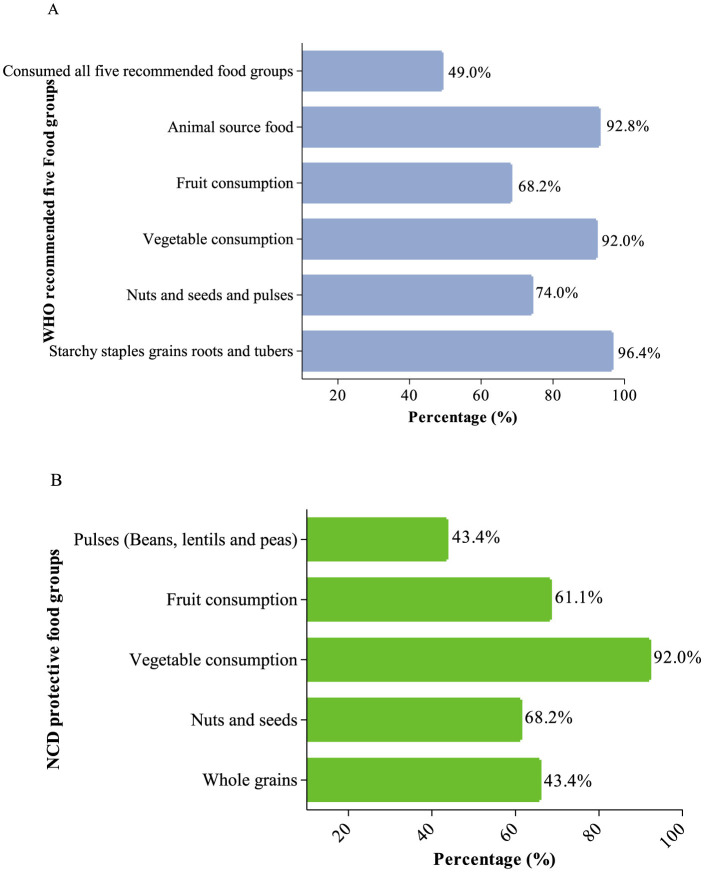
Proportion of consumption of foods from **(A)** the WHO-recommended five food groups and **(B)** NCD-protective food groups.

Almost 50% of the participants ate all-five WHO-recommended food groups (shown in Figure 1). Consumption of vegetables was more than 90% while < 68.2% consumed fruits. More than 90% consumed animal source foods. Participants had a mean score of 4.23 (0.94) for all-five WHO-recommended food groups. Regarding consumption of foods from the NCD-protective food groups, 61.1% consumed nuts and seeds and 43.4% consumed pulses (shown in Figure 1). The mean (SD) NCD protect score was 4.22 (2.01) out of a maximum score of 9.

**Figure 2:**
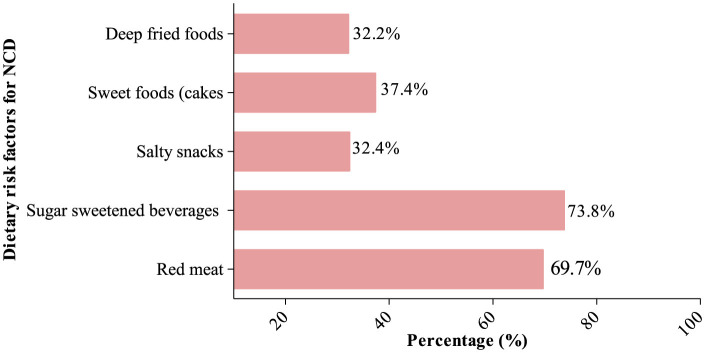
Proportion of consumption of NCD-risk food groups.

Concerning the consumption of foods that could increase one's risk of NCDs, 37.4% consumed sweet foods, 32.2% deep-fried foods and 19.1% consumed processed foods (Shown in Figure 2). Mean NCD-risk scores were 2.89 (2.06) out of a maximum score of 8. Notably, 88% (*n* = 741) of the participants ate at least one NCD-risk food. Participants had a mean (SD) GDR score of 10.33 (2.17) out of a maximum score of 18.

Normality test, as shown in Table 3, showed that the data were not normally distributed.

**Table 3:** Normality test using Shapiro and Kolmogorov-Smirnov.

**Variable**	**Kolmogorov-Smirnov** ^**a**^			**Shapiro-Wilk**		
	**Statistic**	**df**	***P*** **-value**	**Statistic**	**df**	***P*** **-value**
NCD-protect scores	0.102	842	< 0.001	0.97	842	< 0.001
FGDS score	0.100	842	< 0.001	0.973	842	< 0.001
all-5-recommended food	0.283	842	< 0.001	0.768	842	< 0.001
NCD-risk score	0.176	842	< 0.001	0.922	842	0.000
GDR score	0.101	842	< 0.001	0.979	842	0.000

^a^Lilliefors' significance correction.

### Comparison between socio-demographic characteristics and consumption of NCD-protective foods and the WHO-recommended five food groups

As shown in Table 4, there was a moderate positive correlation between NCD-protective food scores and food group diversity scores and with WHO all-five recommended food groups. Strong positive relationships between the NCD-protective score and both the FGDS (*r* = 0.76, *P* < 0.01) and all-5 score (*r* = 0.69, *P* < 0.01). Moderate correlations of the NCD-protective score with the GDR score (*r* = 0.42, *P* < 0.01) and an inverse correlation with the NCD-risk score (*r* = −0.42, *P* < 0.01), supporting their conceptual distinction. Positive intercorrelations among GDR, FGDS, and all-5 scores (*r* = 0.24–0.32, all *P* < 0.01), reflecting overlapping yet unique constructs of dietary adequacy. Age associations were generally weak but significant for the NCD-protective (*r* = 0.094, *P* < 0.01), GDR (*r* = 0.100, *P* < 0.01), and all-5 scores (*r* = 0.064, *P* < 0.05), suggesting slight increases in diet quality with older age.

**Table 4:** Correlation between age and the five diet quality indicators.

**Variable**	**NCD-protect scores**	**NCD-risk scores**	**GDR score**	**FGDS score**	**all-5 scores**
Age	0.094^**^	−0.014	0.100^**^	0.050^*^	0.064^*^
NCD-protective score		0.293^**^	0.423^**^	0.763^**^	0.688^**^
NCD-risk score			−0.420^**^	0.387^**^	0.288^**^
GDR score				0.243^**^	0.323^**^
FGD Score					0.689^**^

^*^The correlation is statistically significant at the 0.05 level. ^**^The correlation is statistically significant at the 0.01 level.

Table 5 shows the mean rank scores among socio-demographic characteristics and the consumption of NCD-protective foods, FGDS, and all-5 WHO-recommended food groups. NCD protect scores, FGDS, and all-5 WHO-recommended food groups differed significantly by ethnicity, marital status, and employment status.

**Table 5:** Comparison between socio-demographic characteristics and scores for NCD-protective foods, food group diversity, and WHO-recommended five food groups.

**Variable**	**NCD-protective food scores**	***P*-value**	**NCD-risk scores**	***P*-value**	**FGD Score**	***P*-value**	**all-5 food group scores**	***P*-value**	**GDR**	***P*-value**
**Sex**		0.959		0.130		0.904		0.972		0.187
Female	417.12		406.37		416.60		417.26		427.28	
Male	417.98		431.32		418.61		417.80		405.35	
**Ethnicity**		< 0.001		0.240		< 0.001		< 0.001		< 0.001
Mole-Dagbani	445.56		426.76		439.89		439.86		438.26	
Akan/Ewe/Gas	276.42		387.36		315.48		313.79		325.38	
Others	442.99		431.87		426.51		430.90		425.00	
**Educational level**		< 0.001								< 0.001
No education	462.47		385.55	0.014	446.47	< 0.001	428.16	< 0.001	479.54	
Primary	503.77		447.55		480.66		455.57		472.19	
Middle/JHS	493.74		466.58		489.16		491.47		436.87	
Secondary	472.75		458.58		458.61		474.37		447.82	
Tertiary	359.09		405.71		374.69		378.26		380.05	
**Marital status**		< 0.001		0.004		< 0.001		0.001		0.001
Married/cohabiting/living with a partner	473.44		438.64		461.18		449.14		446.69	
Divorced/separated/widowed	409.38		302.96		373.81		412.79		478.01	
Never married/single	364.16		411.18		380.56		390.17		387.09	
**Employment status**		< 0.001		0.399		0.001		0.009		0.197
Employed	439.01		417.80		433.75		427.76		420.65	
Unemployed	367.86		403.58		376.72		386.80		398.77	

### Multiple linear regression analysis of the association between demographic factors and diet quality indicators

Table 6 shows the multiple linear regression analysis of demographic factors associated with the five diet quality indicators. Across the diet quality indicators, increasing level of education was significantly associated with increased scores for NCD-protective foods, FGDS, GDR, all-5 WHO-recommended food groups, except for NCD-risk foods. Being married/cohabiting was positively associated with higher scores for NCD-protective foods, NCD-risk foods, and FGDS.

**Table 6:** Multiple linear regression analysis of demographic factors associated with diet quality indicators.

**Variable**	**Adjusted coefficients (95% CI)**	***P*-value**	***R* squared (Adjusted *R*. squared)**
**NCD-protective foods**			0.11 (0.10)
Intercept	3.84 (2.64–5.03)	< 0.001	
No education	0.51 (0.05–0.97)	0.028	
Primary	0.70 (0.16–1.24)	0.012	
Middle/JHS	0.58 (0.10–1.05)	0.018	
Secondary	0.72 (0.35–1.08)	< 0.001	
Tertiary	1		
Married/cohabiting/living with a partner	0.73 (0.01–1.45)	0.047	
Divorced/separated/widowed	0.27 (−0.55–1.08)	0.521	
Never married	REF		
Employed	0.26 (−0.04–0.55)	0.091	
Unemployed	REF		
Akan/Ewe/Gas	−0.32 (−0.87–0.23)	0.262	
Mole-Dagbani/Gonjas	−1.21 (−1.84 to −0.57)	< 0.001	
Others	REF		
Age	−0.01 (−0.02–0.01)	0.611	
**NCD-risk foods**			0.03 (0.02)
Intercept	2.73 (1.58–3.88)	< 0.001	
No education	0.08 (−0.36–0.52)	0.717	
Primary	0.30 (−0.22–0.82)	0.261	
Middle/JHS	0.34 (−0.13–0.79)	0.163	
Secondary	0.32 (−0.03–0.67)	0.074	
Tertiary	REF		
Married/cohabiting/living with a partner	0.73 (0.04–1.42)	0.040	
Divorced/separated/widowed	0.41 (−0.37–1.20)	0.302	
Never married	REF		
Employed	0.09 (−0.20–0.37)	0.545	
Unemployed	REF		
AkanEwe/Gas	−0.04 (−0.57–0.50)	0.891	
Mole-Dagbani/Gonjas	−0.29 (−0.90–0.32)	0.355	
Others	REF		
Age	−0.02 (−0.04 to −0.00)	0.025	
**GDR scores**			0.06 (0.05)
Intercept	10.11 (8.88–11.34)	< 0.001	
No education	0.43 (−0.04–0.90)	0.075	
Middle/JHS	0.25 (−0.24–0.74)	0.318	
Secondary	0.40 (0.02–0.77)	0.039	
Tertiary	REF		
Married/cohabiting/living with a partner	0.00 (−0.75–0.75)	0.998	
Divorced/separated/widowed	−0.15 (−0.99–0.70)	0.733	
Never married	REF		
Employed	0.17 (−0.14–0.48)	0.284	
Unemployed	REF		
Akan/Ewe/Gas	−0.28 (−0.85–0.29)	0.338	
Mole-Dagbani/Gonjas	−0.92 (−1.58 to −0.26)	0.006	
Others	REF		
Age	0.02 (−0.00–0.03)	0.111	
**FGDS scores**			0.06 (0.05)
Intercept	5.30 (4.05–6.54)	< 0.001	
No education	0.41 (−0.07–0.89)	0.091	
Primary	0.58 (0.01–1.15)	0.044	
Middle/JHS	0.55 (0.05–1.04)	0.031	
Secondary	0.56 (0.18–0.94)	0.004	
Tertiary	REF		
Married/cohabiting/living with a partner	0.92 (0.17–1.68)	0.017	
Divorced/separated/widowed	0.61 (−0.24–1.47)	0.159	
Never married	REF		
Employed	0.25 (−0.06–0.56)	0.111	
Unemployed	REF		
Akan/Ewe/Gas	−0.15 (−0.73–0.43)	0.603	
Mole-Dagbani/Gonjas	−0.81 (−1.47 to −0.14)	0.017	
Others	REF		
Age	−0.01 (−0.03–0.01)	0.504	
**all-5 WHO-recommended food groups**			0.06 (0.05)
Intercept	3.83 (3.26–4.40)	< 0.001	
No education	0.06 (−0.16–0.28)	0.591	
Primary	0.19 (−0.07–0.45)	0.153	
Middle/JHS	0.23 (0.01–0.46)	0.044	
Secondary	0.33 (0.16–0.50)	< 0.001	
Tertiary	REF		
Divorced/separated/widowed	0.25 (−0.14–0.64)	0.207	
Never married	REF		
Employed	0.10 (−0.04–0.24)	0.171	
Unemployed	REF		
Akan/Ewe/Gas	−0.10 (−0.36–0.18)	0.466	
Mole-Dagbani/Gonjas	−0.42 (−0.72 to −0.11)	0.007	
Others	REF		
Age	0.00 (0.0 to −0.01)	0.471	

REF, reference; CI, confidence interval.

## Discussion

This study assessed five diet quality indicators to characterize the dietary quality of Ghanaian adults from northern Ghana and the associated demographic characteristics concerning adherence to healthy eating recommendations. Participants exhibited moderate dietary diversity (mean FGDS 6.02/10; 96.4% consumed starchy staples, 88.2% meat/fish, and 48.6% dark leafy greens), with half fulfilling all-five WHO food category guidelines (mean 4.23/5). Nuts and seeds (61.1%) and pulses (43.4%) contributed to a mean NCD-protective score of 4.22 out of 9, whereas 88% of individuals ingested at least one NCD-risk meal (mean 2.89 out of 8), namely sweets (37.4%) and fried snacks (32.2%). The average overall diet quality was 10.33 out of 18. In adjusted models, higher education emerged as the most significant predictor of superior diet quality, while Mole-Dagbani/Gonja ethnicity was associated with lower scores across various indicators. Additionally, married or cohabiting status correlated with increased consumption of both protective and risk foods, older age marginally diminished NCD-risk consumption, and employment showed no independent association.

We found that the consumption of vegetables (an NCD-protective food group) during the previous day or night was more frequent and higher than the consumption of fruits, another NCD-protective food group. In addition, while 92.1% of the participants met the Ghana food-based dietary recommendation for the consumption of a variety of vegetables daily, only 61% met the recommendation for daily consumption of a variety of fruits. This corroborates those of previous studies from Ghana, in which the consumption of fruits among Ghanaians has been relatively low (20). It is unsurprising that the consumption of vegetables was common, given that the staple foods (T-Z, banku, jollof, etc.) of the study setting are usually served with vegetable-based soups and/or sauces. Conversely, fresh fruits are more expensive due to the necessity of transporting bananas, oranges, and pineapples from southern regions, and are less accessible year-round. In addition, they are contingent upon seasonal harvests. Our univariate analyses also demonstrated that unemployment (which was employed as a proxy for lower income) was substantially correlated with a lower quality of diet, thereby confirming the importance of affordability in fruit consumption. This has significant public health implications, as low fruit intake increases the risk of NCDs, such as cardiovascular diseases and diabetes (34–37). To address this issue, public health campaigns should raise awareness about the importance of fruit consumption and work to overcome barriers related to cost and accessibility. Policy interventions, such as subsidies for local fruit production and improved distribution networks, are also necessary to make fruits more affordable and widely available, thereby improving overall diet quality among the population. Commendably, the recently launched food-based dietary guidelines for Ghanaians (38) provide separate recommendations for the consumption of fruits and vegetables as part of efforts to increase the consumption of fruits. This finding emphasizes the importance of conducting qualitative studies to explore the barriers and enablers of fruit consumption. Understanding these factors can inform evidence-based approaches in designing nutrition education interventions and messages that promote the daily consumption of fruits.

Consumption of sugar-sweetened beverages and red meats (NCD-risk food groups) was relatively common, as 70% consumed red meats and 74% consumed Sugar-sweetened beverages (SSB) during the previous day and night. These findings are similar to those reported from previous studies conducted in Kumasi and Accra and other parts of sub-Saharan Africa (39). This demonstrates the reality of the nutrition transition and changing dietary patterns of Ghanaians, probably due to the ubiquitous availability of these foods in the Ghanaian food environment. Shops that sell these SSBs are close to most homes and can easily be accessed. SSBs are also relatively cheap, making them affordable. This is corroborated by the findings of Adjei et al. (40), who investigated the healthiness of supermarkets in six selected districts in the Greater Accra Region. Among the 62 supermarkets assessed, the authors found a ubiquitous availability of unhealthy/ultra-processed foods with nine unhealthy foods for every healthy food, such as noodles, cornflakes, processed meats, cake, and cookies (40, 41). The authors added that, in 4/6 districts (with low-to-medium poverty headcount), fresh meat, fish, or poultry were not available in any supermarket. Supermarkets in two districts with low-to-medium poverty headcount designations did not sell fresh fruits or fresh/unsalted canned vegetables. The high prevalence of NCD-risk food groups' consumption highlights the need for targeted interventions to address unhealthy dietary patterns and promote healthier choices among Ghanaian adults.

An important finding of this study was that 51% of the participants did not meet the recommendation for consuming all five food groups typically recommended for daily consumption in food-based dietary guidelines around the world: fruits; vegetables; pulses, nuts, or seeds; animal-source foods; and starchy staples. These five food groups are considered being indicators of a healthy dietary pattern (10, 30, 42). This finding shows that healthy dietary patterns are probably uncommon in the population, making it necessary for the design of interventions to improve the diet quality of adults in the study setting. The characteristic unhealthy dietary pattern of the participants was also consolidated by the mean (SD) NCD protect score of 4.22 (2.01) out of a maximum score of 9, which is below the minimum score of 5. In addition, < 15% consumed foods from at least seven NCD protect food groups.

Although the mean (SD) NCD-risk score of 2.79 depicted a low consumption, there was a notably high prevalence of consumption of SSBs and red meats that constitute NCD-risk food groups. The high consumption of sugar-sweetened beverages and red meats among participants of this study may be because of urbanization, Western dietary influences, aggressive marketing, and association with social status. In urban areas such as Tamale, these products are widely available and appealing due to convenience and taste preferences. The implications for NCD risk are significant: sugar-sweetened beverages increase the risk of obesity and diabetes, while high red meat intake is linked to cardiovascular disease and cancer (10, 42). These findings emphasize the need for targeted interventions, including public health campaigns and policy regulations, to address the growing NCD burden in Ghana.

As has been previously reported (10), we found in this study that GDR scores corresponded negatively with NCD-risk scores but positively with NCD-protective scores. These findings demonstrate that increasing GDR scores were associated with increasing consumption of NCD-protective food groups and less consumption of NCD-risk foods. Although the correlation was weak, the finding suggests that individuals who consumed more NCD-risk foods like processed foods, sugary snacks, and red meats are also consuming more protective foods like fruits, vegetables, and whole grains. This suggests that individuals may not strictly adhere to a single dietary pattern but may balance their intake of unhealthy foods with healthier options. This demonstrates a dual dietary pattern, where people are incorporating both healthy and unhealthy foods into their diet simultaneously, rather than exclusively following either a healthy or unhealthy eating pattern. We thus, suggest that interventions promoting healthy eating should address both the increase in protective foods and the reduction of risk foods. Public health efforts should focus on encouraging a shift away from unhealthy foods to improve overall diet quality and reduce the risk of NCDs.

The mean GDR score of 10.33 (out of a score of 0–18) shows that participants met barely half of the recommendations for a healthy dietary pattern, corroborating further the relatively unhealthy dietary pattern of the study population. The low GDR score highlights that the participants are following relatively unhealthy dietary patterns, consuming fewer protective foods and likely more foods that increase NCD risk, such as processed and sugary foods. This finding is a red flag for public health, as it reflects the participants' insufficient intake of healthy foods necessary for maintaining optimal health and reducing the risk of diet-related diseases. It underscores a potentially rising burden of NCDs in the population due to poor diet quality. The results imply a need for targeted dietary interventions and nutrition education to encourage healthier eating patterns. Efforts should focus on increasing awareness of dietary guidelines and making healthier food choices more accessible and appealing to the population.

We also found that the consumption of NCD-risk foods significantly decreased with increasing age. This finding demonstrates that as participants grew older, they were more likely to follow a healthy dietary pattern and improve diet quality compared to younger participants. The findings indicate that older participants tend to follow healthier diets, suggesting that as individuals age, they may prioritize healthier eating, likely due to greater awareness of health concerns, possibly leading to more conscious efforts to consume protective foods like fruits, vegetables, whole grains, and other nutrient-rich foods. Younger individuals, on the other hand, may have less healthy dietary patterns, which may be more influenced by convenience, modern lifestyles, and food marketing, leading them to consume more processed and unhealthy foods. These findings are consistent with previous studies from Ghana in which the consumption of rice, pasta, meat, and fish, while the consumption of roots, tubers, and plantain was associated with older age (18, 21, 43). Studies from other parts of sub-Saharan Africa (44). These results imply the need for targeted dietary interventions for younger populations to establish long-term healthy eating habits, while ensuring older adults maintain their protective dietary patterns to reduce NCD risks.

Interestingly, we found that education emerged as a critical determinant of dietary patterns. Individuals with lower educational attainment, particularly those with secondary or middle/junior high school levels of education, showed significantly higher consumption of NCD-protective foods and better scores for FGDS and adherence to the WHO-recommended food groups than those with tertiary education. Participants with tertiary education were more likely to follow unhealthy dietary patterns compared to those with lower education levels. These findings align with a study among Spanish adults by Romero et al. (45) but contradict a study from Zimbabwe, where increased schooling improved dietary diversity among women aged 15–49 years (46). Similarly, a UK study reported that higher education was associated with healthier dietary choices than lower education levels (46). Another study in the US found that adults with low levels of educational attainment reportedly have poorer diets (47). Other studies found no significant association between education and diet quality. For instance, a study from Australia did not find any association between educational attainment and diet quality among adult women (47). Thus, the evidence is inconsistent, with varying findings across different settings. The assumption that higher education leads to better diet quality is based on increased awareness and income, enabling access to healthier foods. However, numerous factors, including cultural traditions, food marketing, and food availability and affordability, may be key contributors to diet quality among adults irrespective of their educational level or socioeconomic status (45). Notwithstanding this, in low- and middle-income settings, higher education may correlate with poorer dietary habits due to the nutrition transition where socioeconomic advancement increases access to both unhealthy and healthy foods (48) but those with lower level of education may have access to traditional indigenous diets (49). These disparities underscore the need for context-specific research to better understand the relationship between education and diet quality across diverse populations.

Our finding that individuals in marital or cohabiting relationships were more likely to consume NCD-protective foods and report higher FGDS scores, similar to those of a previous study from Ghana that found an association between dietary patterns and marital status (43). At the same time, married individuals also reported consuming NCD-risk foods, highlighting the dual dietary patterns seen in transitional food environments. This duality suggests that food consumption may not be driven by the healthiness of the diet but rather by a mix of social, economic, and environmental factors. This behavior reflects the broader nutrition transition, where increased access to both traditional, nutrient-dense foods and energy-dense, processed foods influence dietary choices (50). Social and economic factors, such as pooled resources and shared household preferences, might contribute to these patterns, often prioritizing taste and convenience over healthiness (48). These findings highlight the complexity of dietary behaviors in such settings and underscore the need for culturally sensitive, household-level interventions and regulatory measures to promote healthier eating habits and reduce the consumption of NCD-risk foods.

Another important finding of this study was that participants who were employed and earned income reportedly had higher NCD-protective food scores, FGD scores, all-5 recommended food group scores, as well as NCD-risk food group scores. However, these significant associations were not maintained in the multiple linear regression analysis. The fact that these significant associations did not hold in the multiple linear regression analysis indicates that the relationship between employment and these dietary behaviors may be influenced by other factors (such as education and marital status) that were controlled for in the regression model. In other words, once these other variables were considered, the direct effect of employment and income on diet was no longer statistically significant. As such, while employment initially appears to be linked to better diet quality, other factors are likely influencing these outcomes.

Another important finding was that the Mole-Dagbani/Gonja participants were significantly less likely to consume NCD-protective foods, adhere to all-5 WHO-recommended food groups, or report higher FGDS and GDR scores than their counterparts. This finding may likely result from interconnected factors. Socioeconomic constraints, including poverty and food insecurity, limit access to nutrient-dense foods, while cultural dietary norms emphasize staples like millet and sorghum, which lack diversity (21). Geographic challenges, such as harsh climates and limited agricultural productivity, further restrict food availability, compounded by poor market access and infrastructure (51). Structural inequities, such as inadequate resource allocation and nutrition interventions in northern Ghana, worsen these disparities (52). Addressing these challenges requires targeted strategies to improve food access, enhance nutritional education, and address systemic inequities in these communities.

The strengths of this study include the use of a validated DQ-Q to evaluate the diet of the participants (20). This study contributes to the understanding of the diet quality of Ghanaian adults, and it highlights the need for interventions to improve adherence by informing the composition of food-based dietary guidelines to promote healthy eating and diet diversity among Ghanaian adults. The questionnaire has been designed and validated among individuals aged 15 years and older (20) and based on the Ghanaian context, setting and food environment. Arguably, it is the first community-based study with a large sample size that has investigated the diet quality of adults from northern Ghana. The evidence will serve as a platform for the conduct of further studies and to inform the design of interventions to improve the diet of adults in Ghana and in other countries with similar settings.

This study is not without limitations. Our adoption of a cross-sectional design makes it difficult to establish causality. Furthermore, the use of self-reports and recall could make the findings liable to social desirability and recall biases. The findings may also be limited by the seasonality of foods, which was not assessed in the current study. The DQ-Q is limited by its design, in which quantitative information in relation to the amounts of each food item consumed is not collected. In addition, we did not account for confounding factors such as socioeconomic status, income levels, and others that may influence the association between socio-demographic variables and the five diet quality indicators. In addition, the DQ-Q is limited by assessing dietary intake in the last 24 h only or a one-time measurement, which may not be an adequate assessment of usual intake.

## Conclusion

The diet quality indicators pointed to a pattern largely consisting of starchy staples, grains, and tubers. Diets were largely less diversified, and consumption of NCD-protective foods was less frequent. Consumption of NCD-risk foods, such as SSB and red meats, was common. Participants barely met the global recommendations for healthy dietary patterns. Diet quality is shaped significantly by socio-demographic factors, particularly education, marital status, age, and ethnicity. These findings underscore the importance of designing targeted, culturally sensitive nutrition interventions and policies, especially for lower-educated and marginalized ethnic groups, to promote healthier eating patterns and reduce the risk of diet-related NCDs.

## Data Availability

The original contributions presented in the study are included in the article/supplementary material, further inquiries can be directed to the corresponding author.
